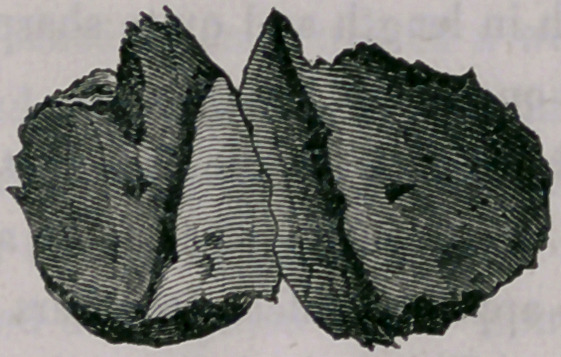# Abstract of the Proceedings of the Buffalo Medical Association

**Published:** 1856-10

**Authors:** Sanford B. Hunt


ART. III.—*Abstract of the Proceedings of the Buffalo Medical Association.*

TUESDAY EVENING, Sept. 2, 1856.

The association met.

Present—The President, Dr. Eastman, in the chair. Drs. Samo, Strong, Devening, Gould, Garvin, Almy, Wilcox, Jeyte, White, Treat, Rochester, Hamilton, Newman, Baker, Dayton, Nichols, Hunt, and Lemon.

The minutes of the preceding meeting were read and approved.

The usual order was suspended, and Drs. William Van Pelt and Levi J. Hamm, of Williamsville, invited to participate in the discussions.

*Prof. White* presented two pieces of the temporal bone removed by the trephine, with the following history:

A young man from Steuben Co., applied to Prof. White on the 12th of June last. Some four years before he had been struck with an axe-helve on the side of the head, just over the left ear. The blow was a severe one, but he did not fall at the time, and during the afternoon succeeding the injury, walked home a distance of eighteen miles. Soon after he exhibited symptoms of compression of the brain, and several physicians being called, the skull was laid bare over the injury, and a depression found. This was raised to place, apparently, by the elevator; but subsequently to the operation epilepsy was developed, and from that time on he was subject to paroxysms, coming on without the usual epileptic aura. These finally became so frequent and severe as to render it dangerous to leave him alone, and he now presented himself for a careful investigation of his case.

On examination no depression was perceptible, but it was considered a fair presumptiom that the internal table was still depressed. The operation of trephining was proposed to him, with a full explanation of the chances of success, as well as the danger to life attending it. Although no promises were made as to success, the patient insisted on the operation, which was performed on the succeeding day. To find the seat of the depression, it was necessary to rely on the statement of an intelligent friend who accompanied him, and had witnessed the previous operation.

The trephine was applied in the usual manner, and the button of bone being raised, it was found that a depression did exist as had been anticipated, but that only a portion of it had been removed. The trephine was again applied, and a second piece removed, which included all of the remaining depression except a small portion which was taken off by the bone forceps. The operation was performed under chloroform.

The wood-cut given below shows the size and character of the depression.

During the succeeding ten days the patient continued to improve steadily, and was able to get about the house. During this time there was no epileptic seizure. At the end of this period he was found one morning in bed, very heavy and comatose, and during the day a large hernia cerebri occurred. From this time the case ran on for a month through various vicissitudes. Lime-water, with firm compression, was the usual dressing; but the solid nitrate of silver was also frequently and thoroughly applied with temporary benefit. At times he became conscious, and more wakeful, but the hernia was not materially diminished by any of the means applied. After a lapse of some weeks, the protruding mass of brain was removed by the knife. It weighed between six and seven drachms. There was again a temporary improvement, which was followed by another protrusion, and finally abscess in the substance of the brain and death.

During all this period no epileptic seizures occurred.

The result of this case, though unfortunate, should not militate against the operation. So far as can be judged, it was successful in its leading object. The unforseen occurrence of hernia cerebri is to be considered as a secondary accident.

*Dr. Treat* said that he had seen two cases of operation by trephining for epilepsy. One of them was a resident of this city, who had a large depression from an injury. Prof. Mott, of New York, operated with partial success. The patient thinks that his paroxysms are less violent than before.

Another case was that of a man who had received a blow from a club, leaving a very marked depression. Two years afterward epilepsy was developed. The operation was performed by an irregular practitioner, and a large piece of bone removed. No benefit whatever followed it.

*Prof. Rochester* said that Prof. Hamilton would recollect a specimen preserved in the college museum. A man was kicked by a horse in Austria, causing a marked depression on the forehead. He was admitted to the hospital for treatment for epilepsy. The case was sent to the surgical ward, but pneumonia coming on, no operation was performed, and the patient was returned to the medical ward, where he shortly after died. A *post-mortem* examination revealed a spiculum of bone projecting perpendicularly into the brain, about half an inch in length and quite sharp, being about two lines in breadth at its base, and one line at its apex.

*Dr. Van Pelt* called attention to the fact that the amount of bone depressed does not inform us as to the effect to be anticipated. Sometimes a large piece produces no apparent effect, in others a small depression is followed by fatal symptoms. This is doubtless owing to locality. For instance, a boy was kicked over the eye by a horse, the toe cork of the shoe striking the bone, causing an elliptical depression. It was evident that the internal table, in such an accident, must be much more depressed than the outer. Nevertheless, a surgeon from the city, who was called in consultation, decided not to trephine, but simply to elevate. In the operation the internal table was detached, and some time after Dr. Van P. was obliged to remove it. Such an accident in another locality would have been fatal.

*Prof. White* described a case of deformity. He had recently removed the second and third toes from the left foot of a young man. Each of them were free from disease, but very large, larger than the great toe, and projecting two inches in advance of it. The nails were large and perfect.

*Dr. Baker* mentioned a curious case of accidental deformity, where, by the result of an injury the middle and ring fingers of *both* hands were closely adherent in their entire length.

*Dr. Hamm* furnished the following case: In 1848 he was called to treat a boy, 11 or 12 years of age, for typhoid fever. The disease was protracted and his emaciation extreme. He finally recovered under a supporting treatment, and became robust; but during this illness he had several large bedsores, sacral and spinal. In June, 1855, the same lad was riding horseback when he felt a soreness near the side of the anus. Two weeks later, riding again, an abscess burst and a fistulous opening continued until January.

Dr. Hamm was then called, and found that the fistula did not communicate with the rectum. It was situated an inch and a-half in front of the anus, and to one side of the mesial line. He laid it freely open, and introduced a tent. On the fourth day thereafter, he felt with a probe something hard in the track of the fistula, but did not remove it till a week later, when it was brought away easily by forceps.

This substance was to all appearance the lower end of the coccyx. If it is so, how should it be found in front of the rectum? Dr. H. could not learn that the boy had had any fall, and if detached by necrosis at the time of the fever, why was it not expelled sooner? The coccyx itself, on examination, seemed to be shorter than natural.

*Prof. White* said that we have some queer products from fistulous openings. Some fifteen years since he had treated a well known gentleman for a fistula near the anus, and removed from it a quantity of hair, looking like the hair of the mane or tail of a horse. All were of course familiar with the occasional presence of hair in ovarian tumors.

*Dr. Treat* once removed an encysted tumor from just beneath the skin, and found it full of hair resembling that of the cow.

*Dr. Baker* once removed a small tumor from the eyelid of a lady—a simple cyst—and found its inner surface studded with small hairs.

*Dr. Van Pelt* described a case, which was to him unusual. It occurred some years since, in the practice of a gentleman with whom he was then associated, and Dr. Van Pelt only saw the closing scene. The patient had had what was considered a remittent fever, and was convalescent, when he suddenly commenced expectorating from the lungs a thin, slightly viscid, inodorous fluid, in color resembling soap-suds. Dr. Van Pelt found him sitting over a pail, and spitting so rapidly as to prevent conversation. He continued to expectorate for about two hours, when he died. During this time he discharged about a gallon of this fluid.

*Prof. Rochester* had seen a case of sporadic cholera. Was called at 2 P. M., on the 29th of August, to a man who had been ill since 11, P. M., of the preceding night. There had been very unusually copious discharges of the rice-water character, without any pain. During Dr. R.'s visit, vomiting of equally well marked rice-water fluid began, and cramps also set in. Large doses of morphine were exhibited, and also laudanum enemata, but as the voice became aphonic, and the extremities became a little cold, he added calomel to the morphine. During six hours, 6 grs. of morphine and ℈ss of calomel were given. The discharges were thus controlled; in eighteen hours the secretion of urine was re-established, and the patient recovered well, with the exception of a severe salivation from the calomel.

Twenty-four hours after the cessation of the discharges, the patient had black, tarry discharges, accompanied with severe tenesmus and tormina. For this Dr. R. prescribed ol. Ricini ℥j, laudanum gtt. xx, which cleared out the bowels, and put an end to the tenesmus.

*Prof. Hamilton* alluded to the use of cathartics in dysentery. He had recently treated four cases, in three of which he gave Epsom salts. In one of those no further treatment was necessary; in the other two the discharges were easily arrested in forty-eight hours after the operation of the salts. In the fourth case, opiates alone were sufficient to control the discharges.

In all these cases, the early discharges preceding the diarrhœa had been profuse, but the action of the salts made it evident, that subsequent to the setting up of the bloody discharges, a further accumulation of fæces had taken place. Dr. H. supposed that the salts had an effect beyond that of a mere removal of irritating contents—a depleting effect, by which they relieved the congestion of the mucous membrane.

*Prof. White* thought that the depleting effect was not desirable. On the contrary, it was his custom to select the mildest possible cathartic, one that should merely relieve the bowels without exhausting his patient. Rochelle salts had been selected by many practitioners, because it was gentler than Epsom. He was himself in the habit of giving the Epsom salts, combined with dilute sulphuric acid, in order to secure a tonic as well as cathartic effect. He supposed that the beneficial effect was due to the removal of irritating contents only, and thought that the gentlest means of securing that end were the best means.

*Prof. Hunt* had long been in the habit of prescribing cathartics in dysentery. He preferred the Rochelle salts as a rapid and painless cathartic, sufficiently active to remove scybala. The occurrence of previous diarrhœa is no contra-indication for its use. In a case treated a day or two before, the dysentery had been ushered in by profuse diarrhœa, yet the cathartic brought away many scybala, and cut short the disease.

(A discussion of some length followed, in which Drs. Van Pelt, Newman, White, Rochester and Hamilton took part. Its length precludes its record. Dr. Newman reminded members that it was not uncommon to see cathartics, if sufficiently drastic, produce dysentery. Dr. Van Pelt thought that cases which had been preceded by copious diarrhœa, could generally be controlled by opiates, but that those preceded by costiveness would require cathartics. Dr. White would reduce the question to a single point, viz., shall we give cathartics with a view to depletory effects, or shall we choose those of the mildest character? Dr. Hamilton sustained his views with much ingenuity. The discussion finally verged off into the theory of evacuants, the action of opiates, the existence of spasm, etc., etc.)

*Dr. Baker* proposed Dr. Wm. Van Pelt, of Williamsville, as an honorary member.

*Dr. Wilcox* proposed Dr. Levi J. Hamm, of Williamsville, as an honorary member.

Both nominations were laid over one month, under the rule.

*Dr. Wm. Howell,* the librarian, having left the city, Dr. Benj. H. Lemon was appointed librarian. *pro tem.*, on motion of Dr. Hunt.

The resolution which had lain over some time, relative to the reports on pneumonia, was called up and read.

"Resolved, That this Association adopt the report of the chairman of the committe on pneumonia."

After a conversational discussion as to the propriety of establishing such a precedent, by Drs. Baker, Hamilton, White, Rochester, Strong, Hunt, Newman and Van Pelt, the resolution was withdrawn by its mover, Dr. White.

And the association then adjourned.

SANFORD B. HUNT, Secretary.

## Figures and Tables

**Figure f1:**